# The Impact of Radiation Dose on CT‐Based Body Composition Analysis: A Large‐Animal Study

**DOI:** 10.1002/jcsm.13741

**Published:** 2025-02-20

**Authors:** Luca Salhöfer, Gregor Jost, Mathias Meetschen, Daniel van Landeghem, Michael Forsting, Denise Bos, Christian Bojahr, Rene Hosch, Felix Nensa, Hubertus Pietsch, Johannes Haubold

**Affiliations:** ^1^ Institute of Interventional and Diagnostic Radiology and Neuroradiology University Hospital Essen Essen Germany; ^2^ Institute for Artificial Intelligence in Medicine University Hospital Essen Essen Germany; ^3^ MR and CT Contrast Media Research Bayer AG Berlin Germany

**Keywords:** animals, body composition, deep learning, radiation, tomography (X‐ray computed)

## Abstract

**Background:**

CT‐based body composition analysis (BCA) enables the extraction of biomarkers from routine CT data. The influence of body composition on the prognosis of different patient groups has been highlighted in recent years. Typically, the segmentation of muscle and fat compartments is performed with a thresholding‐based subsegmentation, which might be influenced by the image noise as a function of radiation dose. This study was performed to investigate the impact of the radiation dose on a fully automated, volumetric CT‐based BCA.

**Methods:**

In this animal study, 20 Göttingen minipigs were subjected to CT scans on six occasions under five different dose settings with gradations compared to the control given in % from volumetric CT dose index (CTDIvol) of the control (5%, 10%, 20%, 40%, control [10.01 mGy]). A database with full dose (FD) and quarter dose (QD) CT scans from The Cancer Imaging Archive served as a human validation cohort. A previously open‐source published and validated BCA network was applied to each scan. The following features were extracted as volumes (mL): bone, muscle, subcutaneous adipose tissue (SAT), intermuscular and intramuscular adipose tissue (IMAT), visceral adipose tissue (VAT) and total adipose tissue (TAT). Statistical significance was assessed by a one‐way ANOVA with Tukey's multiple comparisons or Kruskal–Wallis with Dunn's post‐hoc tests. The correlation between feature volumes in the dose gradations and the control group was analysed using the Spearman or Pearson method.

**Results:**

All BCA features remained consistent up to the 10% group and showed no significant differences compared with the control. In the lowest dose group (5%), there were significant differences concerning the muscle (5% = 1295 mL [211 mL], control = 1338 mL [248 mL]; *p* = 0.032) and VAT volumetry (5% = 353 mL [208 mL], control = 312 mL [204 mL]; *p* = 0.026) with median differences of −3.13% (muscle) and 12.3% (VAT), respectively. Significant and strong positive correlations were observed between all low‐dose groups and the control (*r* > 0.977, *p* < 0.001). The human validation analysis yielded constant volumes for every BCA feature with a strong positive correlation (*r* > 0.933, p < 0.001).

**Conclusions:**

Fully automated BCA maintains consistent results in various low‐dose settings. Significant deviations are only observed after more than 90% dose reduction in the lowest dose settings (5%), which are currently not used in the clinical routine. This large‐animal study demonstrates the consistency of fully automated BCA in different dose settings and may therefore facilitate its integration into the clinical routine.

## 1 Introduction

Computed tomography (CT) is an integral part of modern medical diagnostics. Advancements in machine learning (ML) technologies have further enhanced CT's capabilities, especially concerning precise volumetry of body tissues and organs [1, 2]. Thereby, body composition analysis (BCA) has evolved from manual and semiautomatic segmentation of anatomic structures or regions to an accurate fully automated 3D analysis [3, 4]. In recent years, numerous studies have investigated the association of BCA and overall survival, progression‐free survival, disease severity and numerous other endpoints like prolonged stay in an intensive care unit or the occurrence of major cardiovascular events [5, 6, 7, 8, 9].

For instance, Keyl et al. established muscle‐to‐bone ratio as an independent prognostic indicator for survival among patients with advanced colorectal cancer as individuals exhibiting a higher ratio demonstrated an enhanced probability of survival [9]. In 2021, Choi et al. demonstrated that a reduced, normalized fat volume poses a risk factor for reduced survival in stage I non–small cell lung cancer (NSCLC) [10]. Because this effect was observed in malignant diseases and other chronic and even acute conditions, it underscores the potential significance of these findings for clinical decision‐making [8, 11, 12]. Initial crucial steps towards integration into clinical decision‐making have been taken by implementing the open‐source Body and Organ Analysis (BOA) into the routine radiological practice, as reported by Haubold et al., by adding it as a DICOM node [4]. As a result, reports can be automatically generated directly within routine clinical practice.

Nevertheless, a broad spectrum of distinct scanners and CT protocols are employed in clinical settings. Dose settings, in particular, exert a major influence on the measured Hounsfield units (HU) [13]. For instance, a 50% reduction in the tube current‐time product value leads to an increase in image noise by factor $\sqrt{2}$ [13], resulting in fluctuations between the HU values measured in the CT voxels and the actual density values. In most BCA networks, such as the one studied here, tissue identification is performed by a combination of direct segmentation and HU thresholding‐based subsegmentations [1, 4, 14].

Generally, BCA from low‐dose CT is feasible, as Lee et al. correlated CT‐derived muscle metrics to sarcopenia‐related clinical parameters like handgrip strength [15]. Still to date, only Morsbach et al. have investigated the impact of dose on the segmentation of a single CT slice through theoretical modulation [16]. They found no significant differences in the area of muscle, steatotic muscle and adipose tissue by HU‐thresholding. As ethical and health concerns limit the availability of CT scans of the same individual, in multiple‐dose settings within a single or a few days, no study has so far compared the impact of different CT doses on the performance of fully automated BCA intraindividually. In oncological imaging, a patient's journey encompasses examinations in different institutions, on multiple scanners and in different imaging modalities (e.g., CT and PET‐CT). An international, prospective multicentre study by Smith‐Bindman et al. demonstrated significant variations in dose parameters (e.g., effective dose) between different institutions and even more pronounced between different countries [17]. Besides that interinstitutional variability, a monocentric observational study recorded median differences for the volumetric CT dose index (CTDIvol) of 7.7%, ranging up to a maximum of 69%, for abdominal CT scans on the same scanner with the same protocol [18].

These fluctuations underscore the necessity to foster the longitudinal integration of CT‐based body composition parameters into the clinical workflow by ensuring accuracy regardless of the dose parameters in CT imaging. This paper explores the impact of the applied radiation dose on CT‐based BCA accuracy in a systematic large‐animal study.

## 2 Material and Methods

### 2.1 Animals

Twenty Göttingen minipigs (Ellegaard, Dalmose, Denmark) served as subjects in the study. The study was approved by the State Animal Welfare Committee (Landesamt für Gesundheit und Soziales, Berlin, Germany) and carried out in compliance with the German Animal Welfare Act. All measurements were performed under general anaesthesia. After injection of intramuscular ketamine (15 mL/kg [Pharmacia, Karlsruhe, Germany]), azaperone (2 mg/kg [Stresnil; Elanco GmbH, Bad Homburg, Germany]) and intravenous administration of 7 mg/kg propofol (Propofol‐Lipuro; B. Braun, Melsungen, Germany), the animals were orally intubated and mechanically ventilated with an air‐oxygen mixture. Maintenance of anaesthesia was achieved with an intravenous propofol infusion of 12 mg/kg/h. The vital parameters of the animals were consequently monitored.

### 2.2 CT Imaging

CT scans were performed in the prone position and end‐expiratory ventilation stop during image acquisition on a 192‐slice dual‐source CT scanner (Somatom Force, Siemens Healthineers, Erlangen, Germany) with the following uniform scan parameters: 0.5‐s rotation time, 0.6 pitch, 150‐mm scan length and 90‐kV tube voltage. Tube current was modified among the groups resulting in different tube current‐time products: 20, 35, 70, 140 and 350 mAs (control). Consequently, different dose measurements resulted given as the volumetric and weighted CT dose index (CTDI_vol_, CTDI_w_). Images were reconstructed with 1‐mm slice thickness, a 300 × 300 mm field of view, Br40 kernel and SAFIRE 3 iterative reconstruction. Objective noise measurement was performed by measuring the standard deviation of HU within a 1‐cm^2^ region of interest (ROI) in the erector spinae muscle (Table 1).

**TABLE 1:** Scan parameters of the investigated groups.

Scan parameter	5%	10%	20%	40%	Control
Rotation time (s)	0.5	0.5	0.5	0.5	0.5
Pitch factor	0.6	0.6	0.6	0.6	0.6
Scan length (mm)	150	150	150	150	150
Tube potential (kV)	90	90	90	90	90
Tube current‐time product (mAs)	20	35	70	140	350
CTDIvol (mGy)	0.53	0.96	1.98	4.00	10.01
CTDIw (mGy)	0.32	0.58	1.2	2.4	6.06
DLP (mGy)	7.95	14.40	29.25	60.00	150.15

*Note:* mAs = Milliampere second, s = second, mm = millimetre, kV = kilovolt, CTDIvol = volumetric CT dose index, mGy = milligray, DLP = dose‐length product, HU = Hounsfield Unit. Data are given as median (IQR).

### 2.3 Study Design

Twenty Göttingen minipigs underwent six CT examinations of the epigastrium (repetitions) for a total of 120 examinations. Five noncontrast scans (5%, 10%, 20%, 40%, control) were performed in each examination, for a total of 600 scans. Because of incomplete scans, three exams were removed from the study, resulting in a total number of 117 examinations. For human validation, full dose (FD) and artificially generated quarter dose (QD) CT images from the *Low Dose CT Image and Projection Data (LDCT‐and‐Projection‐data)* dataset of The Cancer Imaging Archive were investigated [19, 20].

### 2.4 Body Composition Analysis

**FIGURE 1:**
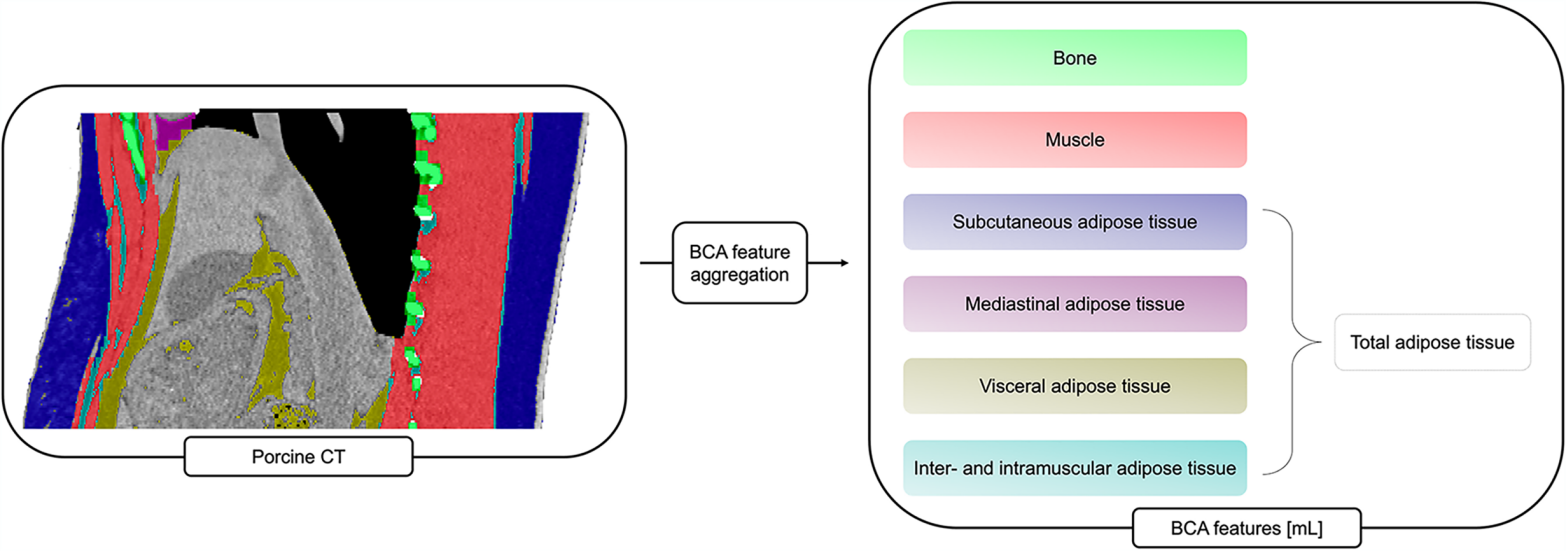
Visualization of the BCA feature extraction. The network detects the different BCA features within the CT scan. The tissues were coded in colour as follows: green: Bone, dark blue: subcutaneous adipose tissue, red: muscle, light blue: intermuscular and intramuscular adipose tissue, yellow: visceral adipose tissue, violet: mediastinal adipose tissue. BCA = body composition analysis, mL = millilitre.

A previously published network that uses a multiresolution variant of the nnU‐Net architecture (BOA) was used to extract the body composition features. This network enables automated tissue segmentation within identified body regions in CT scans with high segmentation accuracy [1, 4]. The following features were extracted and are given as volumes in millilitres (mL), respectively: bone, muscle, subcutaneous adipose tissue (SAT), visceral adipose tissue (VAT), intramuscular and intermuscular adipose tissue (IMAT) and mediastinal adipose tissue (MAT). Total adipose tissue (TAT) volume is the sum of the different fat tissue subtypes (SAT, VAT, IMAT, MAT). Because MAT was not present in every scan, no data concerning that feature is shown. Like many other BCA models, the model employed in this study incorporates a combination of modern segmentation mechanisms and HU‐thresholding for specific tissue types. To identify muscle tissue, the HU threshold is set between −29 and 150. Adipose tissue identification involves thresholding the HU between −190 and −30. For instance, the integration of fat thresholding with intra‐abdominal segmentation leads to the quantification of visceral adipose tissue (VAT) [1] (Figure 1).

### 2.5 Control Segmentation and Validation of the Automated BCA

To assess the reliability of the BOA network, manual segmentations were performed on every fifth slice on 30 CT examinations (5× each dose group) by a first‐year radiology resident with more than 3 years of experience in segmenting CT data in an annotation lab under the supervision of two board‐certified radiologists (9 and 7 years of experience in abdominal imaging). Overlap of the fully automated segmentation and the manual control was assessed by the Dice score. The Dice score is the gold standard of segmentation overlap analysis and a higher score reflects higher agreement [21, 22]:

$$\mathrm{DICE}\;\left(\mathrm{A,}C\right)=\frac{2\times |A\cap C|}{|A|+|C|}$$

A and C represent the fully automatically generated (A) and manually segmented (C) set of images. ∣A∣ and ∣C∣ denote as the respective sample size, while ∣A∩C∣ stands for the size of the intersection between the two groups.

### 2.6 Statistical Analysis

A D'Agostino and Pearson test was used to analyse the normality of the data. Depending on the results, an ordinary one‐way ANOVA with Tukey's multiple comparison tests or a Kruskal–Wallis test with Dunn's post hoc test determined statistical significance. The intraindividual comparison was made up by normalizing the individual segmentations of the investigated groups with lower doses (5%, 10%, 20%, 40%) to the control group in the same scan. Correlation analyses were performed using the Pearson method for normally distributed data and the Spearman method for non‐accnormally distributed data. Data is given as the correlation coefficient (r) with a 95% confidence interval. The Dice score was analysed as an additional metric for the accuracy assessment of the fully automated segmentation of the low‐dose scans compared with the full‐dose scans [21, 22]. For all analyses, the significance level was set at *p* = 0.05. The analyses were conducted and displayed with GraphPad Prism (10.1.1) for MacOS. Normally distributed data is given as mean with standard deviation (± SD) and nonnormal data as median with interquartile range (Q1–Q3) in brackets.

## 3 Results

### 3.1 BCA Model Evaluation

**FIGURE 2:**
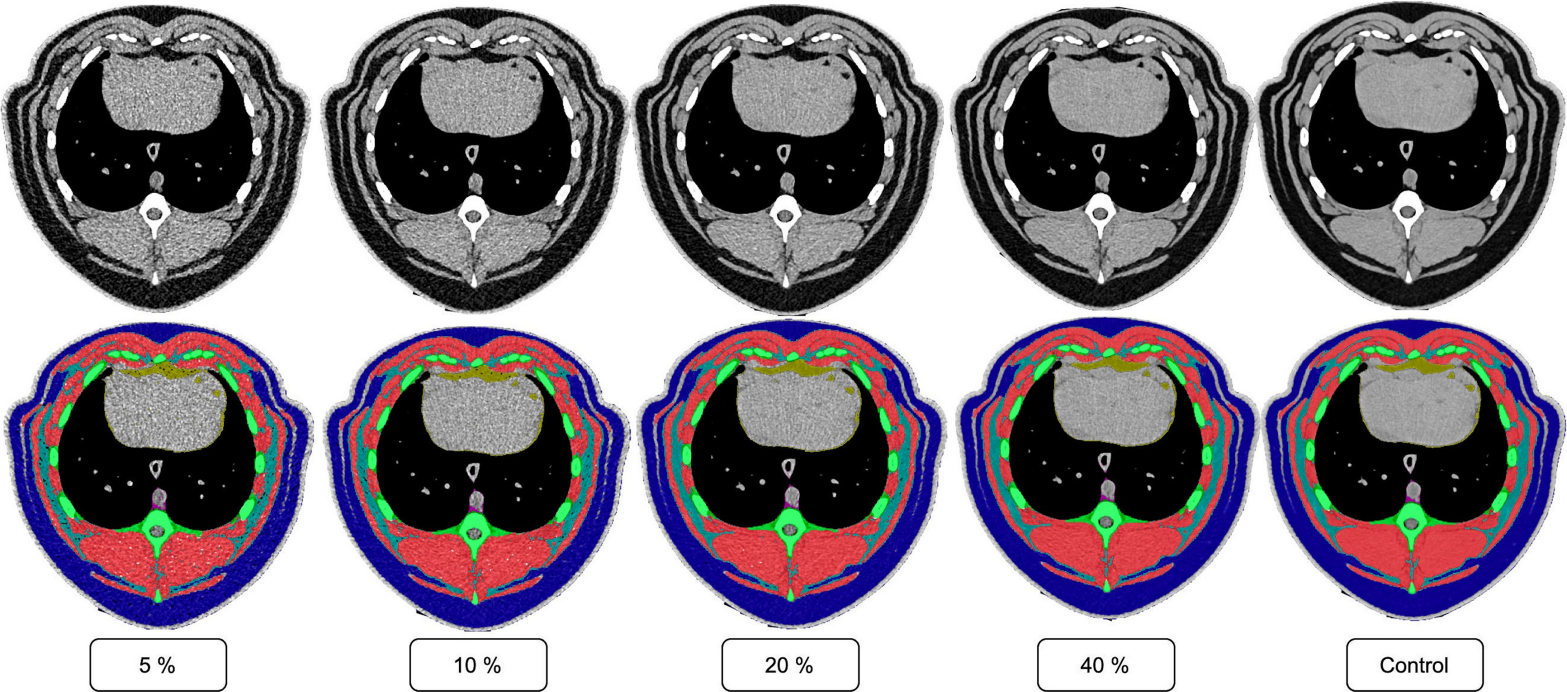
Identical representative CT slice from the different groups, both unsegmented and with segmentation of the BCA features. Axial CT slice on the cardiac level with and without the segmentation of the BCA features in the different dose groups. The tissues were coded in colour as follows: green: Bone, dark blue: subcutaneous adipose tissue, red: muscle, light blue: intermuscular and intramuscular adipose tissue, yellow: visceral adipose tissue, violet: Mediastinal adipose tissue. BCA = body composition analysis.

The overall Dice score for the overlap of fully automated segmentation to manual controls was 0.905 (Dice_ovr_). In the analysis of the subgroups, the following Dice scores were yielded: Dice_5%_ = 0.921, Dice_10%_ = 0.940, Dice_20%_ = 0.912, Dice_40%_ = 0.886, Dice_Control_ = 0.880 (Figure 2).

### 3.2 Image Noise Assessment

**FIGURE 3:**
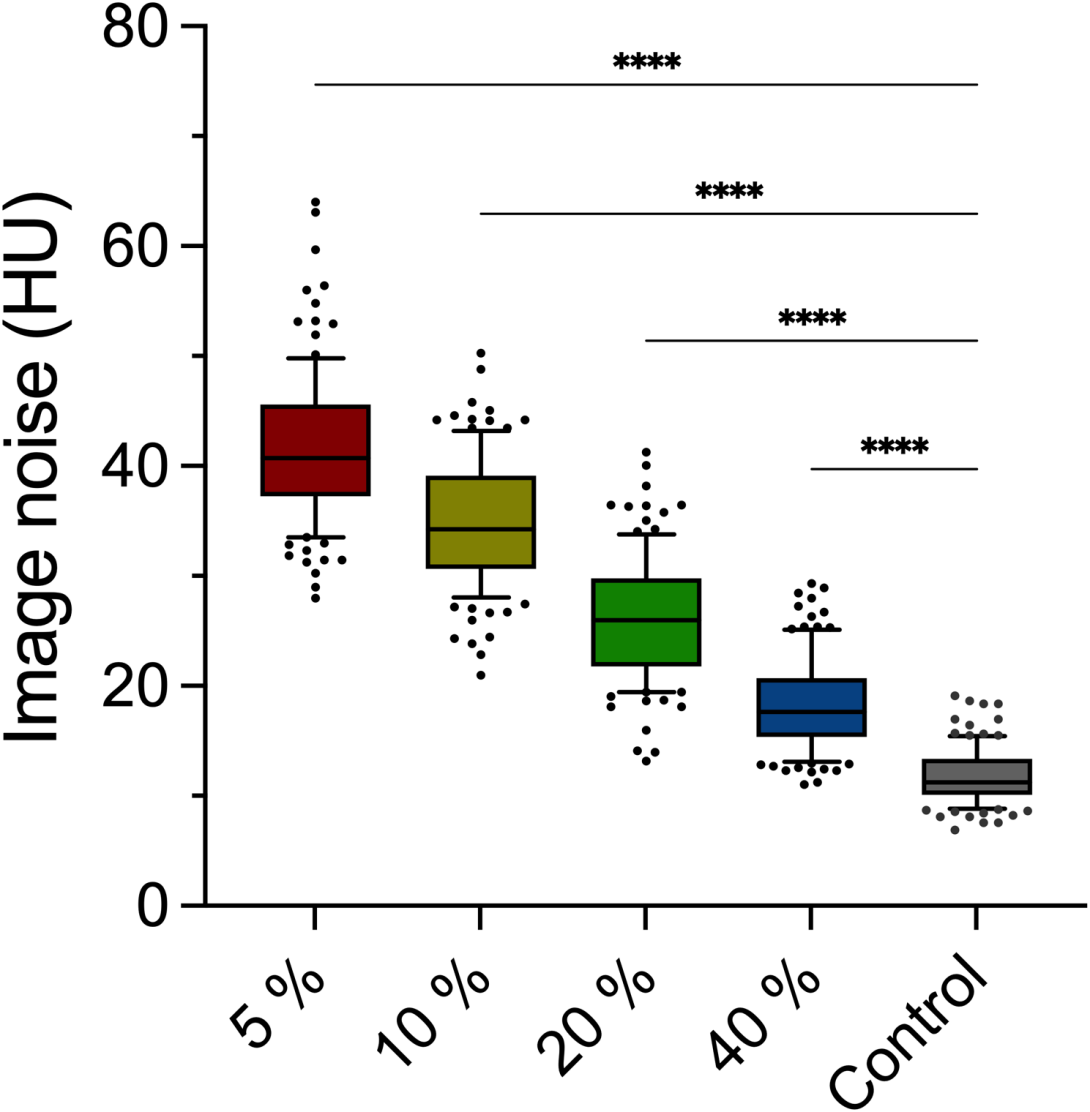
Noise levels of the different dose groups. Every low‐dose group has a significantly higher noise level than the control (11. 3 HU [2.2. HU]). The highest values were observed in the 5% (40.8 HU [8.4 HU]) and 10% (34.2 HU [8.4 HU]) groups. Whiskers represent the 10th and 90th percentile. ****p < 0.0001. HU = Hounsfield unit.

Objective noise level assessment by ROI measurements showed a continuous increase with reduced applied radiation dose. Significant differences exist between the investigated low‐dose groups and the control (11. 3 HU [2.2. HU]). The 5% group had the highest image noise (40.8 HU (8.4 HU); *p* < 0.0001), followed by the 10% (34.2 HU (8.4 HU); *p* < 0.0001), 20% (26.0 HU (8.0 HU); *p* < 0.0001) and the 40% group (17.6 HU (5.4 HU); *p* < 0.0001) (Figure 3).

### 3.3 Quantitative BCA Results

**FIGURE 4:**
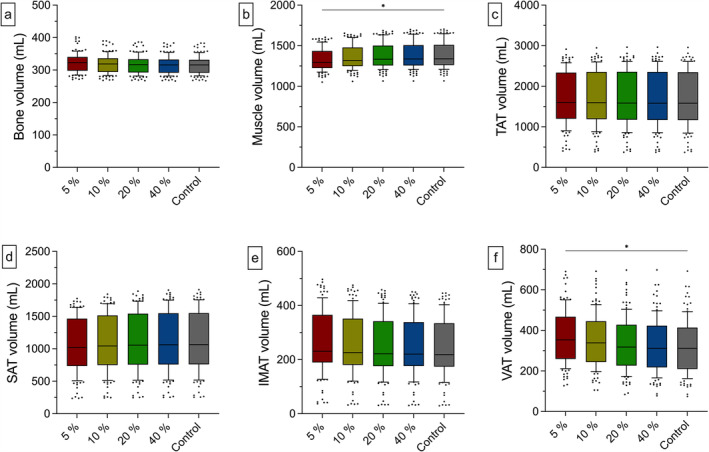
Depiction of the various BCA features under different dose conditions. Compared with the control, a significantly lower muscle volume was segmented in the 5% group (b; 5% = 1295 mL [211 mL], control = 1338 mL [248 mL]; *p* = 0.03). Additionally, significantly higher VAT volumes were segmented in the 5% group compared with the control (f; 5% = 353 mL [208 mL], control = 312 mL [204 mL]; *p* = 0.03). Whiskers represent the 10^th^ and 90^th^ percentile. *p < 0.05. BCA = body composition analysis, mL = millilitre.

**FIGURE 5:**
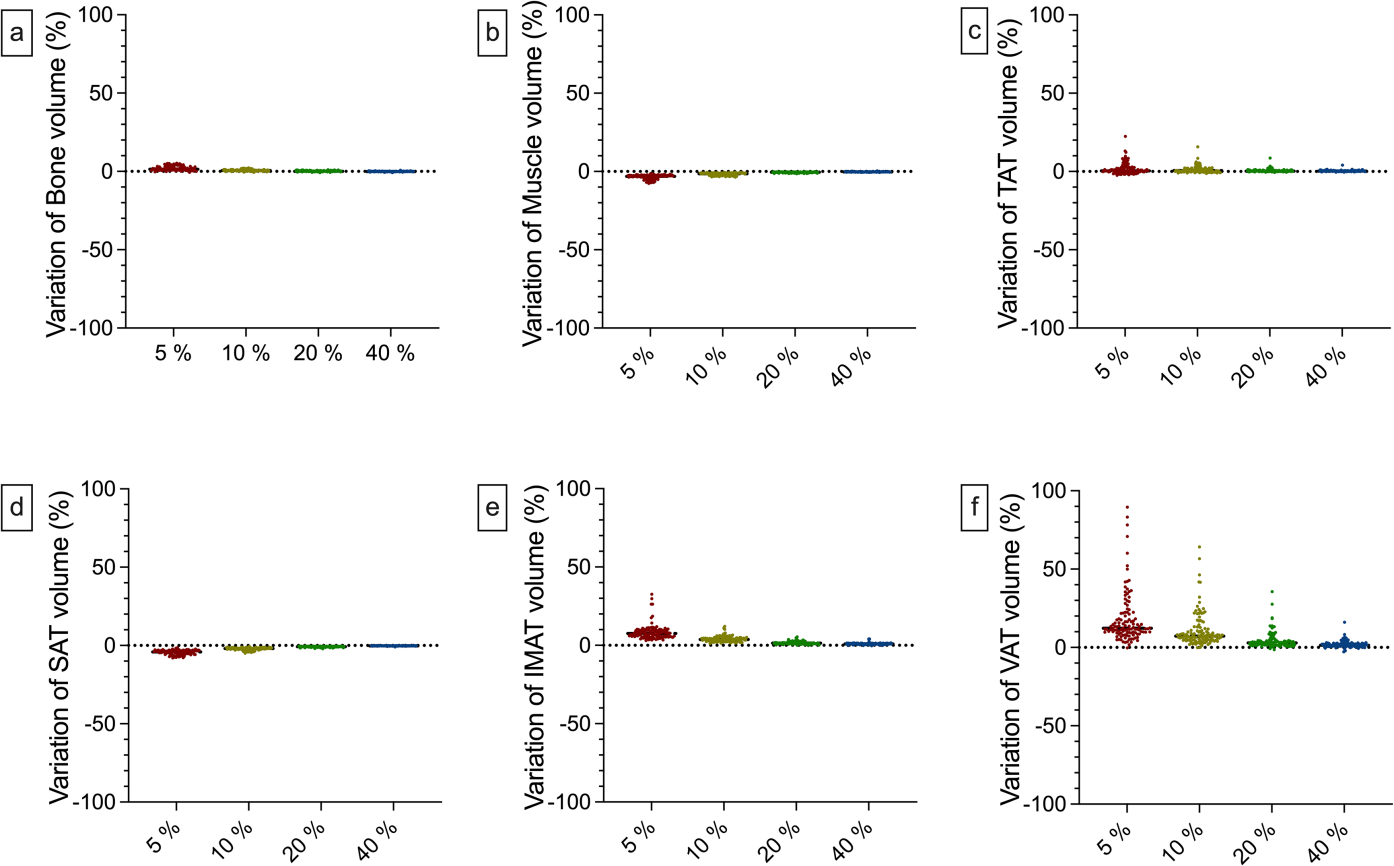
Depiction of the intraindividual variations of the BCA features compared with the control under different dose conditions. No relevant alterations are observed concerning the bone and muscle volumetry (a + b). Concerning the IMAT features, a tendency to higher volumes is observed with a reduced applied dose resulting in a median difference of 7.52% compared with the control (e). For the VAT volume, there is a median difference of 12.3% for the 5% group, 7.3% for the 10% group, 2.9% for the 20% group and 1.4% for the 40% group, compared with the control respectively (f). Dots represent individual values. Horizontal, black and dashed lines stand for the median. BCA = body composition analysis.

The bone volumetry did not differ under the different scan conditions. Between the group with the lowest applied dose (5% = 323 mL [42 mL]) and the control group (316 mL [40 mL]), there were only minor differences. In detail, the 40% group records median volumes that are only 0.003% lower. This difference increases with further dose reduction (20% = 0.153%, 10% = 0.605%) to 1.41% in the 5% group. These results align with the Dice scores for the bone feature, demonstrating consistently high spatial overlap even at the lowest dose (5% = 0.956), further improving with higher dose levels (e.g., 40% = 0.979). Pearson analysis showed a significant and strong positive correlation between the low‐dose groups and the control group (5%: *r* = 0.990 (0.985–0.993), 10%: *r* = 0.998 (0.997–0.999), 20%: *r* = 0.999 (0.999–1.00), 40%: *r* = 1.00 (1.00–1.00); *p* < 0.001). The muscle volumetry in the 5% group yields significantly lower values than in the control group (5% = 1295 mL [211 mL], control = 1338 mL [248 mL]; *p* = 0.03). In the relative intraindividual comparison, there is a median deviation of −3.13% from the 5% group to the control. For the other groups, there are no significant differences to the control (10% = 1318 mL [232 mL], 20% = 1334 mL [247 mL], 40% = 1337 mL [250 mL]). Spatial overlap analysis supports those findings with Dice scores of up to 0.977 (40%). Furthermore, correlation analyses revealed a strong positive link between the low‐dose groups and the control group for the muscle segmentation (5%: *r* = 0.994 [0.992–0.996], 10%: *r* = 0.997 [0.996–0.998], 20%: *r* = 0.999 [0.999–0.999], 40%: *r* = 1.00 [0.999–1.00]; *p* < 0.001). For IMAT volume, only marginal differences are observed between the 10% (226 mL [171 mL]), 20% (222 mL [166 mL]) and 40% group (220 mL [163 mL]) compared with the control (218 mL [161 mL]). Accordingly, the relative median deviations are 0.99% (40%), 1.48% (20%) and 3.84% (10%). By tendency, there is an increased IMAT volume in the 5% group (231 mL [177 mL]) with a median deviation of 7.52%. Despite lower results compared with other features, the Dice score for IMAT remained high and improved consistently with higher dose levels (5% = 0.717, 10% = 0.785, 20% = 0.845, 40% = 0.875). Correlation between the low‐dose groups and the control group was strong and positive (5%: *r* = 0.998 [0.997–0.999], 10%: *r* = 0.999 [0.999–0.999], 20%: *r* = 1.00 [0.999–1.00], 40%: *r* = 1.00 [0.999–1.00]; *p* < 0.001). SAT volumetry shows no significant alterations among the different dose levels, although a tendency to lower volumes can be observed in the 5% (1020 mL (731 mL), median difference: −4.05%) and 10% group (1042 mL (765 mL), median difference: −2%) compared with the control (1065 mL [791 mL]). For all low‐dose groups, correlation analysis showed a strong and positive correlation (5%: *r* = 0.999 [0.999–0.999], 10%: *r* = 1.00 [0.999–1.00], 20%: *r* = 1.00 [1.00–1.00], 40%: *r* = 1.00 [1.00–1.00]; *p* < 0.001) and spatial overlap analysis achieved Dice scores higher than 0.936 (5%). The VAT volumetry in the 5% group yielded significantly higher values compared with the control (5% = 353 mL [208 mL], control = 312 mL [204 mL]; *p* = 0.03) with individual alterations of 12.3% in the median. Without being statistically significant, the 10% group shows a tendency to higher segmented VAT volume (339 mL (201 mL), median difference: 7.28%). In the 20% (318 mL [201 mL]) and the 40% group (312 mL [205 mL]) segmentations showed smaller, individual differences with median deviations of 2.92% for the 20% group and 1.44% for the 40% group, referred to the control. Albeit the lack of statistical significance, there are remarkable differences in the intraindividual comparison in the 10% and 20% groups. VAT segmentations are up to 64.2% (10% group) and 35.7% (20% group) higher, respectively. Visually, there is an increased number of segmented voxels within the gastrointestinal lumen in lower‐dose groups (Figure S1). Still, low‐dose values correlate strongly and significantly with the control group (5%: *r* = 0.977 [0.967–0.984], 10%: *r* = 0.987 [0.982–0.991], 20%: *r* = 0.996 [0.994–0.997], 40%: *r* = 0.999 [0.999–0.999]; *p* < 0.001). For the TAT volume, there is a tendency towards increased volumes with progressively lower doses (5% = 1602 mL [1137 mL], 10% = 1596 mL [1165 mL], 20% = 1586 mL [1180 mL], 40% = 1584 mL [1183 mL], control = 1582 mL [1184 mL]). Compared with the control, there are only slight median intraindividual variations (5% = 0.50%, 10% = 0.59%, 20% = 0.31%, 40% = 0.30%) (Figures 4 and 5). Additionally, correlation analysis showed a strong positive link to the control (5%: *r* = 0.999 (0.998–0.999), 10%: *r* = 0.999 (0.999–1.00), 20%: *r* = 1.00 (1.00–1.00), 40%: *r* = 1.00 (1.00–1.00); *p* < 0.001). For all results of the overlap analyses and a synopsis of the correlation metrics and a graphical illustration, see Tables S1 and S2 and Figure S2.

### 3.4 Human Validation Cohort BCA

**FIGURE 6:**
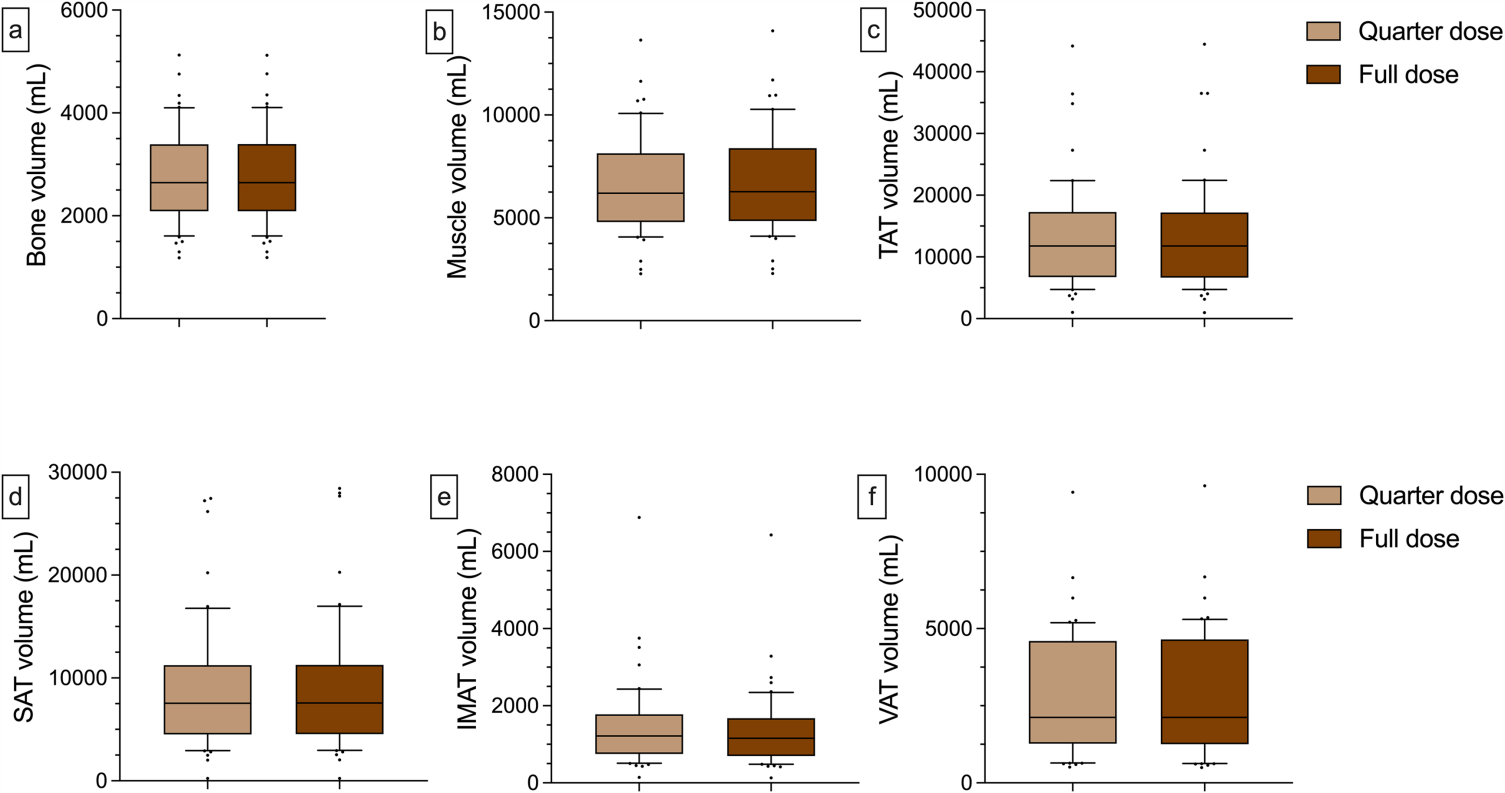
Depiction of the various BCA features in the FD and QD scan. There are no significant differences in BCA between the QD and FD scans. Whiskers represent the 10^th^ and 90^th^ percentile. BCA = body composition analysis, FD = full dose, QD = quarter dose.

The volume of no BCA feature differed significantly between the QD and the FD abdominal CT. Analogous to the porcine experiment, slight tendencies towards a higher IMAT (QD = 1215 mL [1028 mL], FD = 1159 mL [981 mL]) and lower muscle (QD = 6642 mL [2410 mL], FD = 6757 mL [2484 mL]) and SAT volume (QD = 7548 mL [6710 mL], FD = 7579 mL [6725 mL]) were observed in the QD data. Correlation analysis demonstrated significant and strong positive correlations between the QD and FD volumetry (*r* > 0.933, *p* < 0.001) (Figure 6). For the graphical illustrations of the correlation analyses and all metrics, see Figure S3 and Table S3.

## 4 Discussion

Multiple studies investigated the CT‐based BCA at distinct time points (e.g., diagnosis) to overall or progression‐free survival in various acute or chronic conditions as well as in healthy elderly patients [8, 12, 23, 24, 25]. Despite the remarkable number of significant findings, only a few studies have explored the longitudinal development of CT‐based BCA. Lee et al. demonstrated that baseline BCA and the loss of subcutaneous adipose tissue (SAT) within the first 2 months after therapy is an independent risk factor for overall survival [26]. Longitudinal comparison has so far been limited by a presumed lack of transferability of the BCA results under different scan and dose conditions. The goal of this study was to assess the consistency of BCA results under different dose settings in a porcine model and to possibly enhance its comparability.

To our knowledge, no primarily human‐trained BCA network has even been applied to a different species. To achieve valid results, analysis of the segmentation accuracy was paramount. Overall and group‐specific overlap analysis via Dice score revealed excellent segmentation accuracy (e.g., DICE_ovr_ = 0.905). Because segmentation accuracy remained consistent across different dose levels, the slightly lower accuracy compared with human BCA is negligible, as the primary focus of this study was the comparisons between the different groups [1].

To investigate the influence of the radiation dose on the segmentation efficiency, we modified the dose parameters across four investigated groups (5%, 10%, 20%, 40%, control) compared with the control by modulating the tube current‐time product value.

Here, significant deviations were only observed in the lowest applied dose settings (5%) in the muscle (5% = 1295 mL [211 mL], control = 1338 mL [248 mL]; *p* = 0.03) and VAT (5% = 353 mL [208 mL], control = 312 mL [204 mL]; *p* = 0.03) volumetry. In the investigated BCA network, likewise in many others, these features are extracted by HU‐thresholding voxels in a segmented region [4, 14]. Theoretically, the reduction of the tube current‐time product (mAs) leads to an increase in image noise by $\sqrt{\text{factor}}\text{of}\;\mathrm{mAs}-\text{reduction}$ [13]. In the porcine CT examination conducted in this study, there is a progressive increase in image noise compared with the control group, resulting in approximately 3.6‐fold higher noise in the 5% group. This increased image noise is the most plausible explanation for the significant deviations in the lowest dose group. The applied dose in this group is only 5% of the CTDIvol of the control group, and an even smaller fraction compared with the dose reference levels for abdominal CTs without contrast, according to the latest guidelines (e.g., 0.53 mGy [5% group] to 16 mGy [dose reference level of the American College of Radiology recommendations]) [27]. Concludingly, this lacks clinical relevance because of its missing application in practice as the United States and European reference values for CT examinations display remarkably higher CTDI_vol_ compared with the investigated 5% group [27, 28, 29]. Independent of reference values, it is crucial for the comparability of BCA results that no significant deviations occur across different dose levels in the clinical routine. Various studies demonstrate significant variations in dose parameters between institutions and even within the same scanner using identical protocols at the same institution [17, 18]. Additionally, for a better assessment of BCA results, comparison with a healthy, age‐adjusted population is more informative than isolated analysis within the investigated disease groups. Consistency and standardized scanning conditions are essential for reliable integration into reference values, which were established by Tonnesen and Marquardt et al. [30, 31]. The results of our animal study demonstrate the consistency of the analysed features in the applied BCA model, with near‐perfect correlation (e.g., *r* = 0.999 [0.999–0.999] for IMAT [10%]) and excellent outcomes in spatial overlap analysis (e.g., Dice_20%_ = 0.971 for muscle) compared with the control. This indicates that within all clinically acquired CT scans, there is a good comparability of BCA features.

Beyond the implementation in daily care, opportunistic screening BCA, either within the clinical context or CT‐screening programs, would provide an enormous addition as it is possible to stratify for cardiovascular risks [32, 33]. The use of low‐dose CT for thoracic examinations has revolutionized the screening for primary lung cancer [34, 35]. In this context, Xu et al. have already demonstrated added value for BCA on distinct vertebral levels concerning disease‐specific survival, overall survival and cardiovascular mortality [36]. Regardless of whether individual slices are annotated as in Xu et al., or a three‐dimensional scan volume is examined as in other models [4], it remained unclear what impact the applied dose has on the measurements. The data from our study suggest that low‐dose CT protocols, such as those from lung cancer prevention studies, may be utilized for body composition analysis, because the dose parameters (CTDI_w/vol_) in the 10% group, which provided consistent results compared with the control in our animal study, are below the established reference values of cancer screening trials [37, 38]. Because dose values are difficult to translate from our large animal study to humans and within the human species (e.g., due to variations in size and weight), comparing image noise is a sensible approach. The BCA network employed here shows consistent results up to a noise level of 34.2 HU (8.2 HU) in the erector spinae muscle while significant differences only appear at 40.8 HU (8.4 HU) in the 5% group. To support our findings, we validated them using abdominal CTs from the Low Dose CT Image and Projection Data (LDCT‐and‐Projection‐data) dataset of The Cancer Imaging Archive. In this dataset, a quarter‐dose (QD) dataset was generated employing artificial noising based on full‐dose (FD) CT scans. Analysis of all BCA features revealed no significant differences and showed excellent positive correlation between QD and FD scans (e.g., muscle: QD = 6642 mL [2410 mL], FD = 6757 mL [2484 mL], *r* = 0.999 [0.998–0.999]), further reinforcing the results of our animal study. Sooner or later, further dose reduction will necessitate a re‐evaluation of automated BCA in the ultra‐low‐dose range. Correcting features generated by HU‐thresholding with a coefficient may become necessary in ultra‐low‐dose conditions.

In the volumetry of the VAT, unlike the other parameters, there is not only a more distinct median deviation but also some remarkable outliers of up to: 18.9% (40% group), 37.1% (20% group), 64.3% (10% group) and 89.8% (5% group). Visually, more endoluminal voxels within the stomach and intestine were segmented (Figure S1). This observation aligns with known findings from the literature that, in fat‐isodense Hounsfield Unit thresholding within the abdominal cavity, faeces and other endoluminal density changes can also be annotated [39]. Therefore, future studies should eliminate the gastrointestinal lumen from VAT analysis.

Despite the many interesting findings, some limitations need to be addressed. In our study, the influence of image noise on BCA was analysed by manipulating the milliampere‐second value. All other scan parameters (e.g., field of view and reconstruction kernel) remained constant, leaving the influence of those factors unclear. Further studies should evaluate this impact, particularly since Morsbach et al. proposed that BCA‐derived indices, such as the skeletal muscle index, may vary depending on the tube potential [40]. Additionally, only noncontrast scans were assessed in this study and the influence of contrast medium application remains to be investigated.

Finally, this systematic large‐animal study with a human validation analysis demonstrates the feasibility of applying fully automated BCA in different settings with reduced doses and higher image noise. Therefore, this study might be a cornerstone to facilitate BCA integration into the clinical routine. Additionally, this study may pave the way to yield opportunistic imaging parameters by BCA from screening examinations at low doses.

## Ethics Statement

The manuscript does not contain clinical studies or patient data. The study was approved by the State Animal Welfare Committee and carried out in compliance with the German Animal Welfare Act.

## Conflicts of Interest

The study was performed in cooperation with the Bayer AG. Hubertus Pietsch and Gregor Jost are employees of the Bayer AG. The remaining authors declare no conflict of interest concerning the presented study.

## Supporting information


**Figure S1:** Representative CT slices without and with VAT segmentation.
**Figure S2**: Correlation analysis of BCA features between low‐dose and Control volume.
**Figure S3**: Correlation analysis of BCA features between QD and FD data for the human validation.
**Table S1**: Correlation metrics of porcine BCA analysis.
**Table S2**: Dice Score of the different dose levels compared to the Control.
**Table S3**: Correlation metrics of human validation BCA analysis.
