# Supplementary material for: The Impact of Radiation Dose on CT‐Based Body Composition Analysis: A Large‐Animal Study
Source: J Cachexia Sarcopenia Muscle. 2025 Feb 20;16(1):e13741. doi: 10.1002/jcsm.13741 (PMC11842463; doi:10.1002/jcsm.13741)
Supplement: Supplementary file 1 — Figure S1: Representative CT slices without and with VAT segmentation. Figure S2: Correlation analysis of BCA features between low‐dose and Control volume. Figure S3: Correlation analysis of BCA features between QD and FD data for the human validation. Table S1: Correlation metrics of porcine BCA analysis. Table S2: Dice Score of the different dose levels compared to the Control. Table S3: Correlation metrics of human validation BCA analysis. [file JCSM-16-e13741-s001.docx]

# Supplementary Material


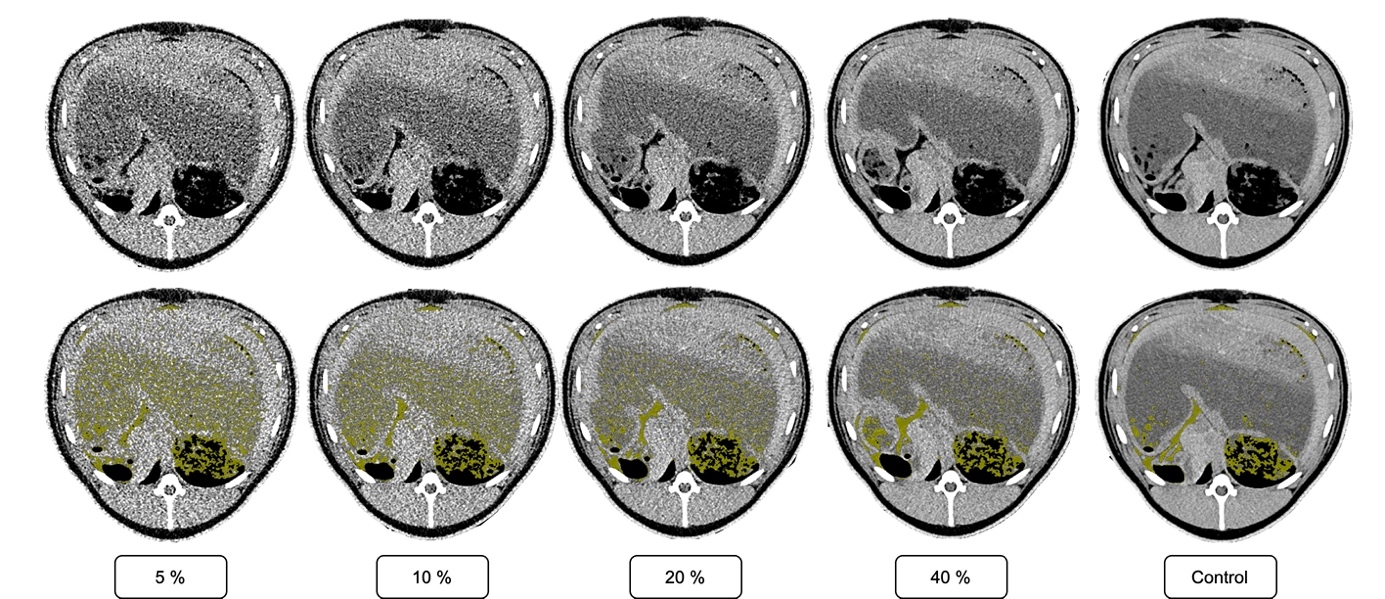


### Fig. S1 Representative CT slices without and with VAT segmentation

#### An exemplary demonstration of VAT segmentations (yellow) as an intra-individual comparison of the different groups in one animal: 5% = 433.38 ml, 10% = 375.44 ml, 20% = 310.28 ml, 40% = 265.68 ml, Control = 228.66 ml

VAT = visceral adipose tissue, ml = milliliter


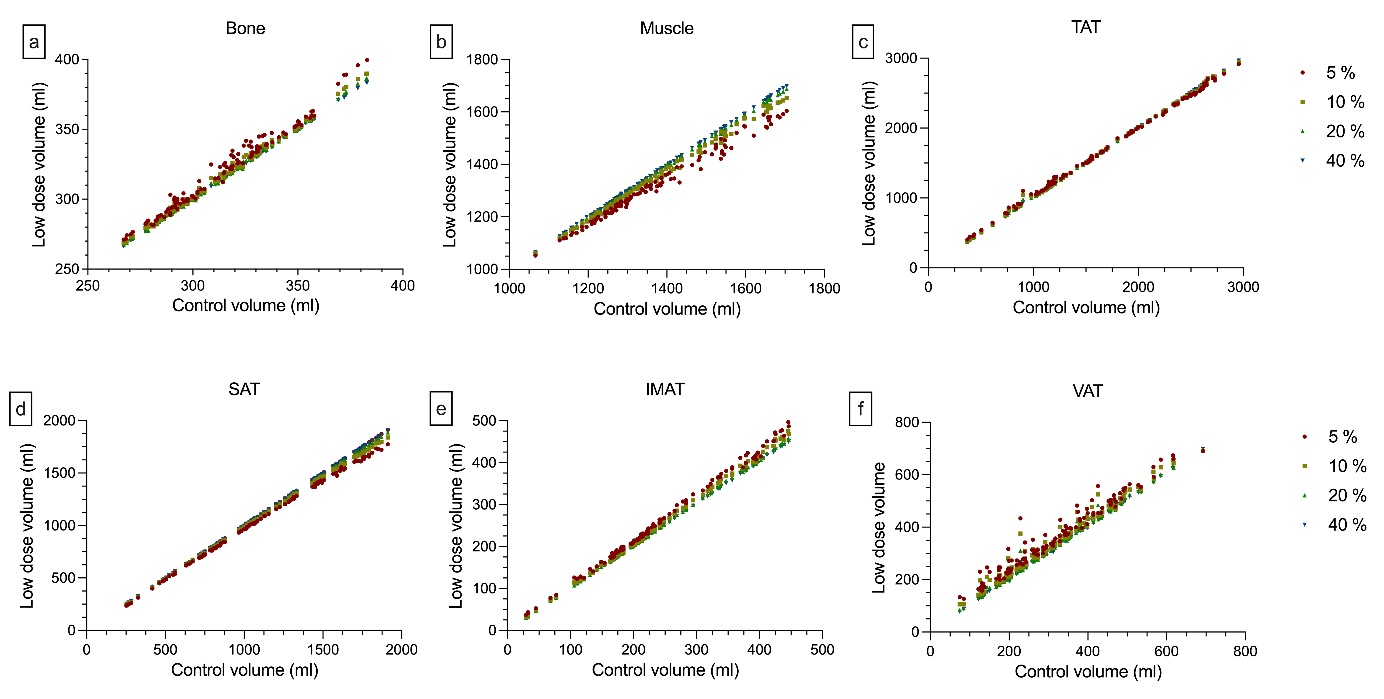


### Fig. S2: Correlation analysis of BCA features between low-dose and Control volume

#### **All investigated BCA features showed a strong and significant correlation between the low dose volumes and the Control volume. By trend, correlation decreased with further dose reduction (e.g. Muscle volume 5 %: r = 0.994, 10%: r =0.997, 20%: r = 0.999, 40%: r = 1.00; p < 0.001).**

**TAT = total adipose tisssue, SAT = subcutaneous adipose tissue, IMAT = inter- and intramuscular adipose tissue, VAT = visceral adipose tissue, ml = milliliter**

**
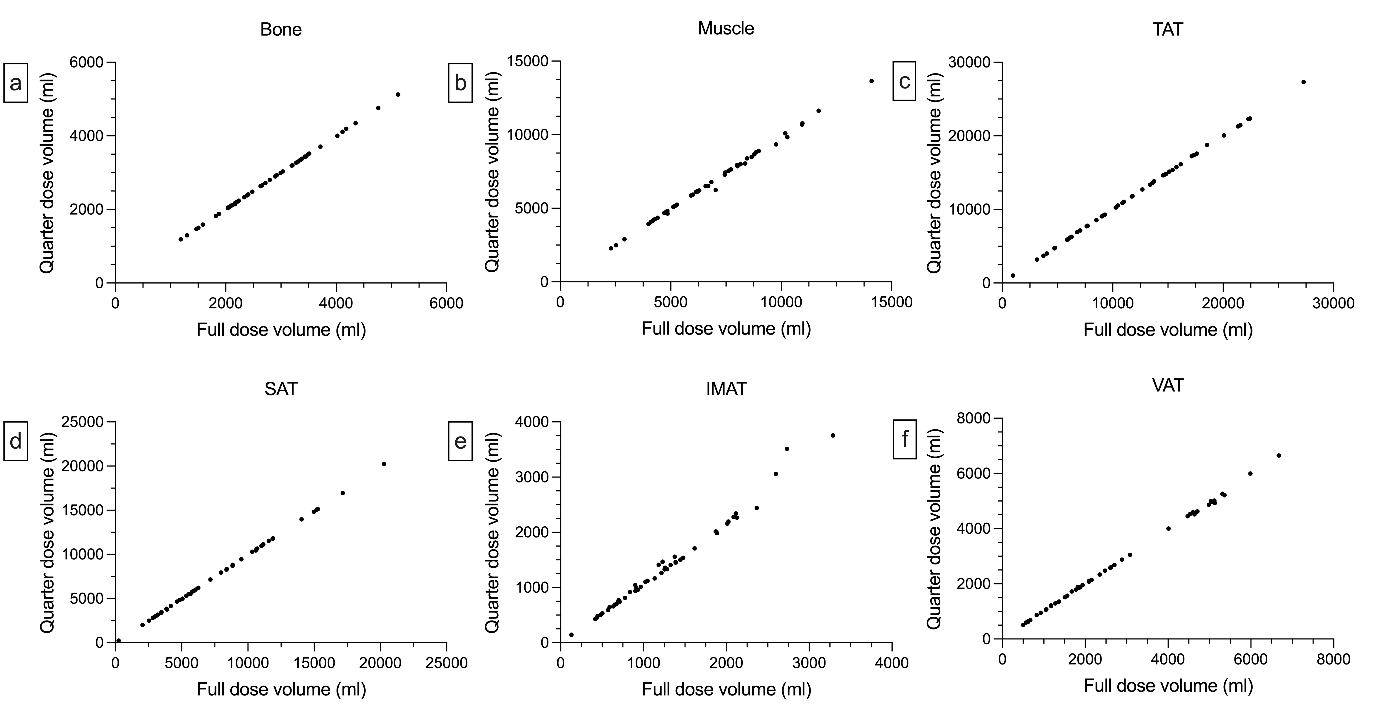
**

### Fig. S3: Correlation analysis of BCA features between QD and FD data for the human validation

#### **All investigated BCA features showed a strong and significant correlation between the low dose volumes and the Control volume. By trend, correlation decreased with further dose reduction (e.g. Muscle volume r = 0.999 (0.998 - 0.999); p < 0.001).**

**TAT = total adipose tissue, SAT = subcutaneous adipose tissue, IMAT = inter- and intramuscular adipose tissue, VAT = visceral adipose tissue, ml = milliliter, QD = quarter dose, FD = full dose.**

### Table S1: Correlation metrics of porcine BCA analysis

| **Feature** | **5%** | **10%** | **20%** | **40%** |
| --- | --- | --- | --- | --- |
|  |  |  |  |  |
| **Bone** | **0.990 (0.985 - 0.993)** | **0.998 (0.997 - 0.999)** | **0.999 (0.999 - 1.00)** | **1.00 (1.00 - 1.00)** |
| **Muscle** | **0.994 (0.992 - 0.996)** | **0.997 (0.996 - 0.998)** | **0.999 (0.999 - .100)** | **1.00 (0.999 - 1.00)** |
| **SAT** | **0.999 (0.999 - 0.999)** | **1.00 (0.999 - 1.00)** | **1.00 (1.00 - 1.00)** | **1.00 (1.00 - 1.00)** |
| **IMAT** | **0.998 (0.997 - 0.999)** | **0.999 (0.999 - 0.999)** | **1.00 (0.999 - 1.00)** | **1.00 (0.999 - 1.00)** |
| **VAT** | **0.977 (0.967 - 0.984)** | **0.987 (0.982 - 0.991)** | **0.996 (0.994 - 0.997)** | **0.999 (0.999 - 0.999)** |

#### **SAT = subcutaneous adipose tissue, IMAT = inter- and intramuscular adipose tissue, VAT = visceral adipose tissue. Data is given as the correlation coefficient (r) with a 95% confidence interval.**

### Table S2: Dice Score of the different dose levels compared to the Control

| **Feature** | **5%** | **10%** | **20%** | **40%** |
| --- | --- | --- | --- | --- |
|  |  |  |  |  |
| **Bone** | **0.956** | **0.968** | **0.976** | **0.979** |
| **Muscle** | **0.939** | **0.956** | **0.971** | **0.977** |
| **SAT** | **0.936** | **0.956** | **0.971** | **0.976** |
| **IMAT** | **0.717** | **0.785** | **0.845** | **0.875** |
| **VAT** | **0.647** | **0.712** | **0.778** | **0.801** |
| **Overall** | **0.820** | **0.861** | **0.896** | **0.910** |

#### **SAT = subcutaneous adipose tissue, IMAT = inter- and intramuscular adipose tissue, VAT = visceral adipose tissue**

### Table S3: Correlation metrics of human validation BCA analysis

| **Feature** | **QD/FD** |
| --- | --- |
|  |  |
| **Bone** | **1.00 (1.00 - 1.00)** |
| **Muscle** | **0.999 (0.998 - 0.999)** |
| **SAT** | **1.00 (0.999 - 1.00)** |
| **IMAT** | **0.993 (0.987 - 0.996)** |
| **VAT** | **0.998 (0.997 - 0.999)** |

#### **SAT = subcutaneous adipose tissue, IMAT = inter- and intramuscular adipose tissue, VAT = visceral adipose tissue, QD = quarter dose, FD = full dose. Data is given as the correlation coefficient (r) with a 95% confidence interval.**
